# Supplementary material for: Stochastic dynamics of Francisella tularensis infection and replication
Source: PLoS Comput Biol. 2020 Jun 1;16(6):e1007752. doi: 10.1371/journal.pcbi.1007752 (PMC7304631; doi:10.1371/journal.pcbi.1007752)
Supplement: Attachment — Submitted filename: FT_PLoS_replies_18_12_2019.pdf [file pcbi.1007752.s001.pdf]

Summary of changes and replies to referees.

We thank the reviewers for reading our manuscript and are pleased to have made the minor improvements they sought.

Referee 1

I read and assessed this manuscript as a general disease ecologist with a mathematical perspective. I would first like to congratulate the authors on a nicely written manuscript; the biology of the system and the model were well-described and highly accessible.
The authors model the growth of intracellular and extracellular populations of Francisella tularensis following initial host exposure. The primary improvement on previous models of this system is to model cell rupture as a catastrophic event; by subjecting intracellular bacterial populations to a birth-death-catastrophe process, their model introduces stochasticity and strong variation into the growth of the total bacterial population.
The authors compare their mathematical results with results from an agent-based model and experimental time series data. They describe the dynamics of infected cells and the bacterial population and find that β (intracellular birth rate) is a critical parameter regulating the system. The manuscript is a nice demonstration of how within-cell processes affect between-cell transmission and overall infection dynamics. I have a few suggestions that I believe will improve the overall impact of the paper.

1. Table 1 would be much better as a figure; it contains key empirical data that support the modeling results. As it is presented, the scientific notation makes it difficult to immediately and easily compare among treatments, organs and time periods. Plotting the average value along with SD would eliminate the need to show values for each of the six individuals. Also, why aren't the low dose data presented here?

   As suggested, we have created a figure consisting of three plots that show the high, medium and low dose data for each organ. Geometric means and geometric standard deviations have been included. The original table has been moved to the supplementary material.

2. Lines 114-117 and 284-286: The authors state that the ratio of macrophages to extracellular bacteria is sufficiently high in the first 72 hours that co-infection of a macrophage does not occur. However, bacterial migration to other tissues does occur during this time period, which requires a high number of extracellular bacteria (escaping macrophages). It seems that, if extracellular bacteria populations are sufficiently high for migration, there should be an increasing probability of co-infection (indeed Fig. 11 shows that infection rates and migration are positively correlated). It would be helpful to understand the densities and interactions of these two players by plotting their populations through time. Seeing the ratio of macrophages to extracellular bacteria should illuminate when migration can occur and whether co-infection is possible.

   Indeed, we agree that there is an increasing probability of co-infection as the number of uninfected macrophages decreases with time. However, the agent-based model allows for co-infection of macrophages but suggests this is a rare event, with the analytic expressions for total bacterial counts (that do not assume co-infection) agreeing well with the simulations shown in Fig. 8. Furthermore, large numbers of extracellular bacteria are not required for migration to other tissues. For each independent bacterium, the probability of migration is

approximately 0.04 (Fig. 12), and so we have observed realisations where bacteria are present in other organs after a couple of hours due to a macrophage rupturing earlier than normal.

3. `In figures 9 and 10 (at high, medium and low initial doses) the modeling results demonstrate earlier arrival of bacteria into non-lung tissues than the empirical work supports. In lines 370-380 this is attributed to bacterial detection, but it could also be that the model results are off. Can this section describe any limitations to the model that might be leading to higher than observed migration rates?`

   We have added some discussion of this. In particular "On the other hand, the experimental data does not allow us to determine the mode of migration from the lung to other organs, nor to place tight constraints on the associated timescales."

4. The discussion could also better tie the results into the implications for this disease. The authors open the manuscript by discussing tularemia as an extremely infectious pathogen and potential biothreat agent, but the discussion never interprets the results in this context. Three important conclusions of the work are that β has strong effects on the system, that the time between rounds of bacterial division is 6 hours, and that there is a reasonably high rate of migration from lung to additional tissues. How do these results matter for progression of tularemia in humans? The empirical results are from a mouse-model, is there work on human tularemia that supports the conclusions?

   We have added some discussion of this. In particular "Data from human or primate infection is even more rare than murine data. In silico models serve as a bridge between animal and human research, with the advantage that human pharmacokinetic and pharmacodynamic parameters can be directly applied. Mathematical models, suitably developed and validated, can provide a suite of tools to estimate the result of experiments, inform their design and extrapolate to humans."

5. I found the discussion of the work to be fairly narrow and focused only on the Francisella system. The birth-death-catastrophe process is a novel and exciting component of this work. The authors mention that this process is important for other intracellular pathogens, but those other systems are never actually described or discussed. Given the insights gained here, how might the birth-death-catastrophe process inform disease dynamics in other systems?

   We have added some discussion of this. In particular "Our modelling approach is applicable to other intracellular pathogens, such as *Salmonella enterica*, and Anthrax, where models must also consider germination of spores. In epidemic models, birth and death events describe the infection and recovery of animals within a disease reservoir; an analogue of a catastrophe event is the "spillover" of disease into a human population.signalling events,used to represent reversible phosphorylation events initiated by the binding of a ligand; dissociation of the ligand terminates the signal."

6. Lines 34-38: Is this there any empirical evidence supporting the load-dependent hazard rate? (It seems biologically reasonable, but empirical support would strengthen this key assumption)

We have found additional evidence to support this assumption. A previous study observed that intracellular replication of *F. tularensis* bacteria in murine macrophage-like J774.A1 cells results in dose-dependent cytopathogenicity. Cells with greater numbers of intracellular bacteria released greater amounts of LDH, an indicator of membrane disruption.

7. `Line 59-61: Clarify here that extracellular bacteria in the blood were also measured.`

   We have confirmed that bacterial counts in the blood were measured, but also included a comment indicating why a compartment representing the blood was not included in the mathematical model: "Although bacterial counts have also been measured in the blood, the numbers were small enough (< 10 CFUs) that this compartment can been neglected from the model."

8. `In all figure legends, it would be helpful to redefine the parameters and variables as much as possible so that the reader doesn't have to flip back to earlier text/tables to interpret them. The authors have done this in some, but not all, figure legends.`

   We have modified figure legends accordingly. In figures showing model predictions using the results of the Bayesian inference, we have made it clearer that an ensemble of parameters sets have been used and thus there is no single parameter set to report.

Referee 2

`Carruthers et al. constructed stochastic models for the infection and replication dynamics of Francisella tularensis. The authors then compared model simulation with experimental data and quantified key parameters for the life cycle of the bacteria in vitro. Francisella tularensis is an important pathogen because of its virulence and transmissibility. However, our quantitative understanding of the life-cycle of the pathogen is very limited. This may hinder the development of effective drugs or vaccines. Thus, the work addresses an important question. The mathematical analysis is rigorous. The methodology is novel and can potentially applicable for other pathogens. The manuscript is well written. I recommend publication once the following concerns are addressed.`

1. `First, the paper emphasizes very much on descriptions of the mathematical methodology and the model. However, the implications of the model and how the model results would be useful for addressing biological questions or clinical questions, for example, developing therapeutics, are not well discussed. I think one or two paragraphs of discussion of how the results of this study relates to the implications of the work would substantially increase the relevance of the work.`

   We have added paragraphs in the introduction and discussion. In particular, although antibiotics such as levofloxacin and ciprofloxacin are commonly used to treat tularemia, their success relies on early administration which is often made difficult given the non-specific symptoms. the current model could be used alongside pharmacokinetic and pharmacodynamic models to describe the concentration of antibiotic in each organ and the effect it has on the bacteria.

2. `Second, for a lot of the parameters in the model (e.g. Table 2), it is not clear how those parameter values were derived; or whether it is consistent with what we know about the life cycle of the pathogen. I understand the precise values may be hard to get due to lack of experimental studies, but it would be important`

`to test the sensitivities of the conclusions of the work against uncertainties in these parameter values.`

More information about the *rationale* for particular parameter values has been included, in Figure legends and in main text. Results of a global sensitivity analysis are included in Fig. 9. For parameters whose values are identified from existing literature (ϕ and μ_E), the low sensitivity indices suggest that changes in their value would have little effect on the output of the model. We therefore feel that the model predictions and inference are robust to any uncertainty in these parameters values.

3. `Third, in the introduction (Page 2), the authors claim that classical models ‘assume that each infected cell, independently, releases infectious particles at a constant rate’. The statement is OK given most of the previous models indeed made those assumptions. However, this statement also ignores a recent work by Koelle, et al. Virus Evolution 2019; 5 (2), vez018, where this assumption can be relaxed. I think that work is directly related to the model developed in this manuscript and it is important to include it in the introduction of the literature.`

   We have included this reference, and modified the introduction, thank you.
